# (-)-Epicatechin Is a Biased Ligand of Apelin Receptor

**DOI:** 10.3390/ijms23168962

**Published:** 2022-08-11

**Authors:** Andrés Portilla-Martínez, Miguel Ángel Ortiz-Flores, Eduardo Meaney, Francisco Villarreal, Nayelli Nájera, Guillermo Ceballos

**Affiliations:** 1 Laboratorio de Investigación Integral Cardiometabólica, Instituto Politécnico Nacional, Ciudad de Mexico 11340, Mexico; 2 School of Medicine, University of California San Diego, La Jolla, CA 92093, USA

**Keywords:** APLNR, β-arrestin, bias ligand, (-)-epicatechin, molecular dynamics

## Abstract

(-)-Epicatechin (EC) is part of a large family of biomolecules called flavonoids and is widely distributed in the plant kingdom. Several studies have shown the beneficial effects of EC consumption. Many of these reported effects are exerted by activating the signaling pathways associated with the activation of two specific receptors: the G protein-coupled estrogen receptor (GPER), a transmembrane receptor, and the pregnane X receptor (PXR), which is a nuclear receptor. However, the effects of EC are so diverse that these two receptors cannot describe the complete phenomenon. The apelin receptor or APLNR is classified within the G protein-coupled receptor (GPCR) family, and is capable of activating the G protein canonical pathways and the β-arrestin transducer, which participates in the phenomenon of receptor desensitization and internalization. β-arrestin gained interest in selective pharmacology and mediators of the so-called “biased agonism”. With molecular dynamics (MD) and in vitro assays, we demonstrate how EC can recruit the β-arrestin in the active conformation of the APLN receptor acting as a biased agonist.

## 1. Introduction

(-)-Epicatechin (EC) is part of a large family of biomolecules called flavonoids. It is widely distributed in the plant kingdom; it can be found mainly in green tea, grapes, and cocoa. Several studies have shown that the intake of EC induces beneficial effects on the skeletal muscle and the cardiovascular system, reducing potential risk factors, including arterial hypertension, endothelial dysfunction, damage to skeletal muscle’s sarcomeric structure, and mitochondrial malfunction by promoting mitochondrial biogenesis [1,2,3,4,5]. Many of the reported EC-induced effects are associated with the activation of intermediaries of specific signaling pathways, such as the mitogen-activated protein kinases (MAPKs), Akt, and AMPK pathways, which may depend on transmembrane receptor activation. Recently, using molecular and functional approaches, we demonstrated that EC interacts with two receptors, the G protein-coupled estrogen receptor (GPER), a transmembrane receptor, [5,6], and the pregnane X receptor (PXR), a nuclear receptor [7]. EC-induced GPER activity induces vasodilation through increases in nitric oxide synthase activity [6]. It also induces mitochondrial biogenesis [5]. EC-induced PXR activity relates to the modulation of cytochromes CYP450 (Cyp3a11) and increases the expression of myogenin [7]. However, neither receptor stimuli seem to be associated with the EC-induced effects found in skeletal muscle. 

In the recent past, working on the isolation and characterization of the possible interaction of PXR and EC, we employed an affinity chromatography column exposing EC. Interestingly, several proteins from skeletal muscle were isolated. 

In this work, we analyze the isolated proteins, searching for any possible receptor that allows us to explain the EC-induced effects in skeletal muscle; with this goal in mind, we employed several approaches, including mass spectrometry for the identification of any possible protein/receptor interacting with EC; molecular dynamics (MD) to analyze binding characteristics and how the macromolecule changes its conformational state, and the affinity [8,9,10] in the presence of EC, in silico; and binding characteristics and functional pathways in vitro. 

## 2. Results

### 2.1. APLNR Isolation in Mouse Skeletal Muscle

A total of 415 peptides were isolated and identified, corresponding to 157 proteins. Five proteins were identified as receptors: apelin receptor, cation-dependent mannose-6-phosphate receptor, glutamate receptor ionotropic (NMDA 2A), glutamate receptor ionotropic (NMDA 2D), and gamma-aminobutyric acid receptor subunit beta-2.

The cation-dependent mannose-6-phosphate receptor is a receptor associated with transport/endocytosis [11], glutamate receptor ionotropic (NMDA 2A) and glutamate receptor ionotropic (NMDA 2D), and gamma-aminobutyric acid receptor subunit beta-2 associate with ion transport [12,13,14,15]. The reported effects on skeletal muscle (Table 1) do not explain the EC-induced effects on SKM. Interestingly, stimuli of the apelin receptor, a G protein-coupled transmembrane receptor [16], induce several similar effects to EC (Table 2). Based on this comparison, we next explored the possibility of EC and APLNR interaction using several approaches.

### 2.2. Akt Phosphorylation in C2C12 Assay

**Figure 1:**
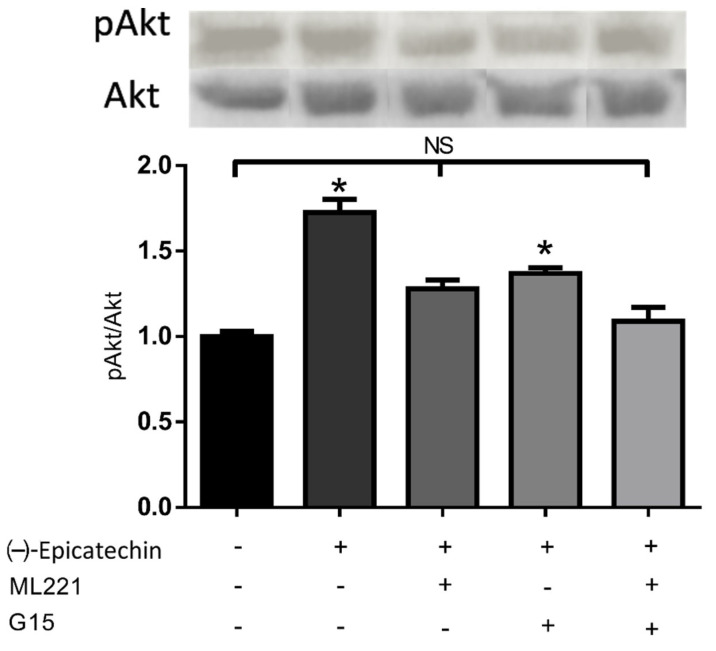
Acute effects (30 min) of EC on upstream Akt activation and the inhibition of these effects with G15 (GPER antagonist), ML221 (APLNR antagonist), and the combination of both. Each Western blot is representative of three independent experiments. Data are expressed as mean ± SD (*n* = 3). * = *p* < 0.05, NS (nonsignificant).

In Figure 1, the EC-induced Akt phosphorylation and the effect blockage of phosphorylation in the presence of GPER antagonist G15, the APJR antagonist ML221, and both antagonists are shown simultaneously. The EC-induced effects on pAkt/Akt ratio (activation) are significantly different from the control group (*p* < 0.05). G15 and ML221 partially block the EC-induced effect. Only the combination of both antagonists seems to block the EC-induced effect completely.

### 2.3. β-Arrestin Recruitment Assay

The protein analysis strongly suggested an active APLNR and epicatechin binding. To prove this possibility, we analyzed the binding characteristics of EC and the natural agonist apelin-13 to APLNRR using the PathHunter^®^ eXpress GPCR kit that explores, in vitro, the β-arrestin recruitment induced by the agonists. The data were analyzed using the operational model for partial agonists proposed by Black and Leff in 1983 [37] (GraphPad Prism version 8). This operational model is derived from the observation that the relationship between receptor occupancy and response is hyperbolic. The receptor’s affinity for the ligand (KA) and its efficacy (tau) were calculated. The results showed a KA and tau (20.8 and 21.8, respectively) for EC binding and a lower value for KA and tau with the combination of EC and ML-221 (0.53 and 1.53, respectively) (Table 3). Apelin-13, the natural agonist, was used as a positive control and for the comparative, ML-221 (5-[(4-Nitrobenzoyl)oxy]-2-[(2-pyrimidinylthio)methyl]-4H-pyran-4-one) an antagonist acting inhibiting cAMP and β-arrestin pathways [38].

**Figure 2:**
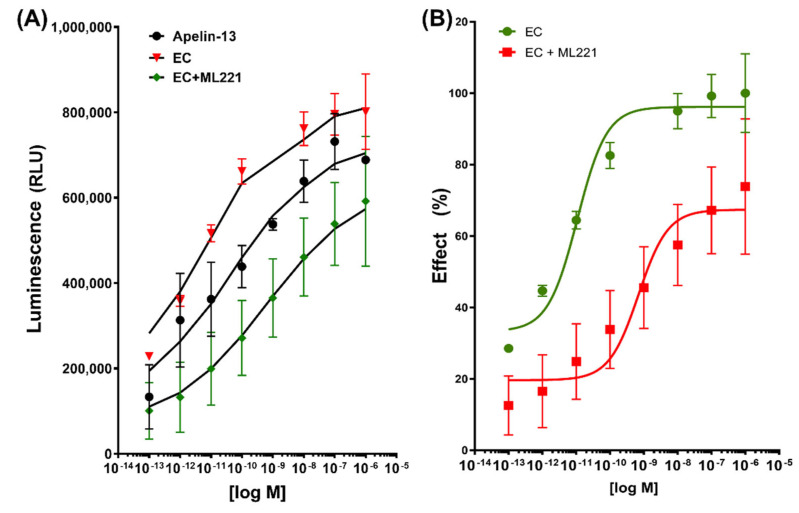
(**A**): Nonlinear regression with the specific binding with hillslope model for apelin-13, (-)-epicatechin (EC), and the mixture with its antagonist ML221. Relative luminescence units (RLU) are plotted on the *y*-axis, and data are expressed as mean S.E.; (**B**) dose–response curve of normalized data taking the EC effect as 10%.

The recruitment of β-arrestin is subject to the ligand binding to the receptor. In this model, chemiluminescence is a specific response to ligand binding. The maximum binding to the receptor (*Bmax*) was calculated, normalizing the values with apelin-13 as 100%; the results showed 101.1% for EC and 91.6% for EC + ML221 (*Bmax* non-normalized values reported in Table 3). On the other hand, the concentration necessary to reach half of the maximum equilibrium binding (*K_d_*) was 2.508 × 10^−11^ M for apelin-13, 1.755 × 10^−12^ M for EC, and 1.012 × 10^−9^ M with EC + ML221 (Table 3 and Figure 2A).

Taking the maximal EC binding as 100%, when the cells were previously incubated with the ML221 antagonist, the binding of EC was displaced to the right, only reaching a maximal binding of 71.57% compared with EC, an inhibitory concentration 50 (IC50) value of 6.93 × 10^−11^ M, demonstrating the interaction of the antagonist with the receptor and the blockage of the effect of EC (Figure 2B).

### 2.4. G Protein Pathways in EPI-Induced Activation of APLNR

We could not find any EC-induced effects (inhibition) on the cAMP production (data not shown). These results suggest that EC does not act through this pathway.

We decided to explore the possibility of EC binding to APLNR in a biased form, using an in silico approach and analyzing the binding characteristics compared with CMF-019 (potassium 3(S)-{[1-(1-ethyl-propyl)-2-thiophen-2-ylmethyl-1H-benzoimidazole-5-carbonyl]-amino}-5-methyl-hexanoate, a bias molecule agonist).

### 2.5. Molecular Docking

**Figure 3:**
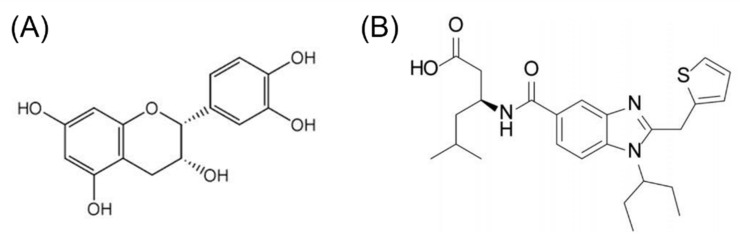
(**A**) (-)-Epicatechin and (**B**) CMF-019 molecular structures.

We analyzed the possible interaction of APLNR with EC and CMF-019 (Figure 3). Both molecules can bind to the receptor and interact at relatively the same site. In the active (aAPLNR) state, both ligands interact with residues Trp85 and Ile109; in addition, EC interacts with residues Tyr182 and Pro292 CMF-019 with residues Tyr88, Arg168, Met183, Tyr264, and Phe291. In the inactive (iAPLNR) state, the ligands do not share residues, EC interacts with Pro292 and Tyr299, and CMF-019 interacts with residues Trp85, Ile109, Arg168, Tyr271, Met288, and Phe291. 

**Figure 4:**
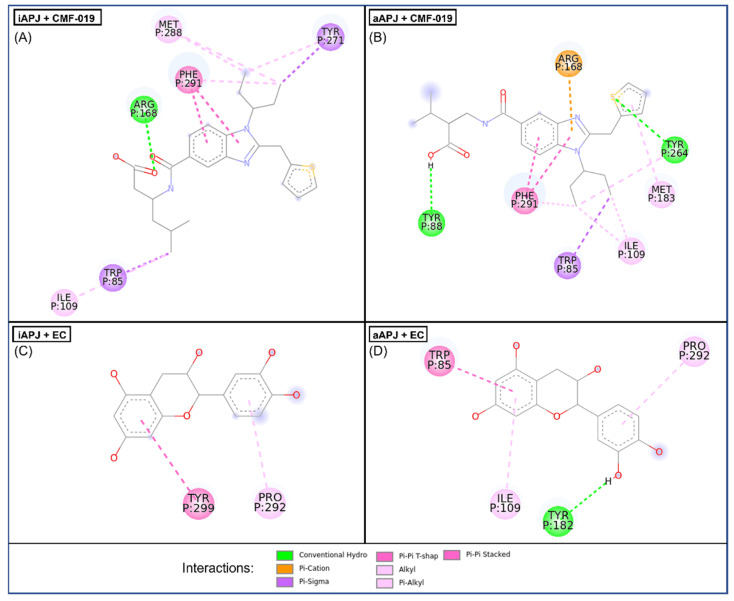
In the four images, we present the binding site and the interactions of the molecular docking results. (**A**) CMF-019 binds to the iAPLNR receptor and shows eight hydrophobic interactions and one polar interaction, of which it is worth highlighting two π-π interactions with the Phe291 residue and the hydrogen bond with the Arg168 residue. (**B**) The CMF-019 binds to the aAPLNR receptor and establishes eight hydrophobic interactions, two electrostatic interactions, and one ionic, of which we can highlight two π-π with the Phe291 residue; two hydrogen bonds, one with Tyr88 and one with Tyr264; and finally, the formation of a π-cation with residue Arg168. (**C**) EC in the inactive conformation of the receptor (iAPLNR) establishes few interactions in contrast to the other three assays, counting only two interactions of a hydrophobic nature with residues Pro292 and Tyr299. (**D**) On the other hand, the EC in the active conformation of the receptor (aAPLNR) establishes four interactions, three hydrophobic and one electrostatic, of which it is worth highlighting the hydrogen bond with Tyr182 and the π-π with Trp85.APJ = APLNR. Images were made with Discovery Studio [39].

**Figure 5:**
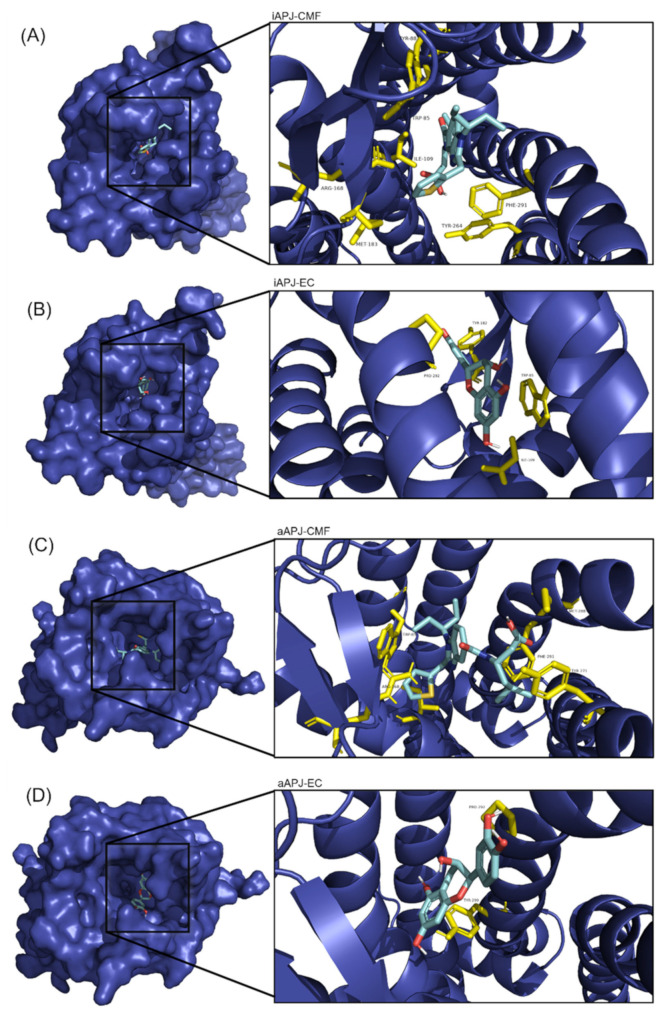
On the left side of each image, the APLN receptor seen from above is shown in a surface representation, and within the cavity (binding site) is the corresponding ligand. A close-up of the binding site is shown to the right of each image, with the amino acid residues in yellow and the ligand in cyan blue. (**A**) The binding site of the CMF-019 in the inactive APLNR conformation. (**B**) The binding site of the (-)-epicatechin in the inactive APLN conformation. (**C**) The binding site of the CMF-019 in the active APLNR conformation. (**D**) The binding site of the (-)-epicatechin in the active APLNR conformation.

**Figure 6:**
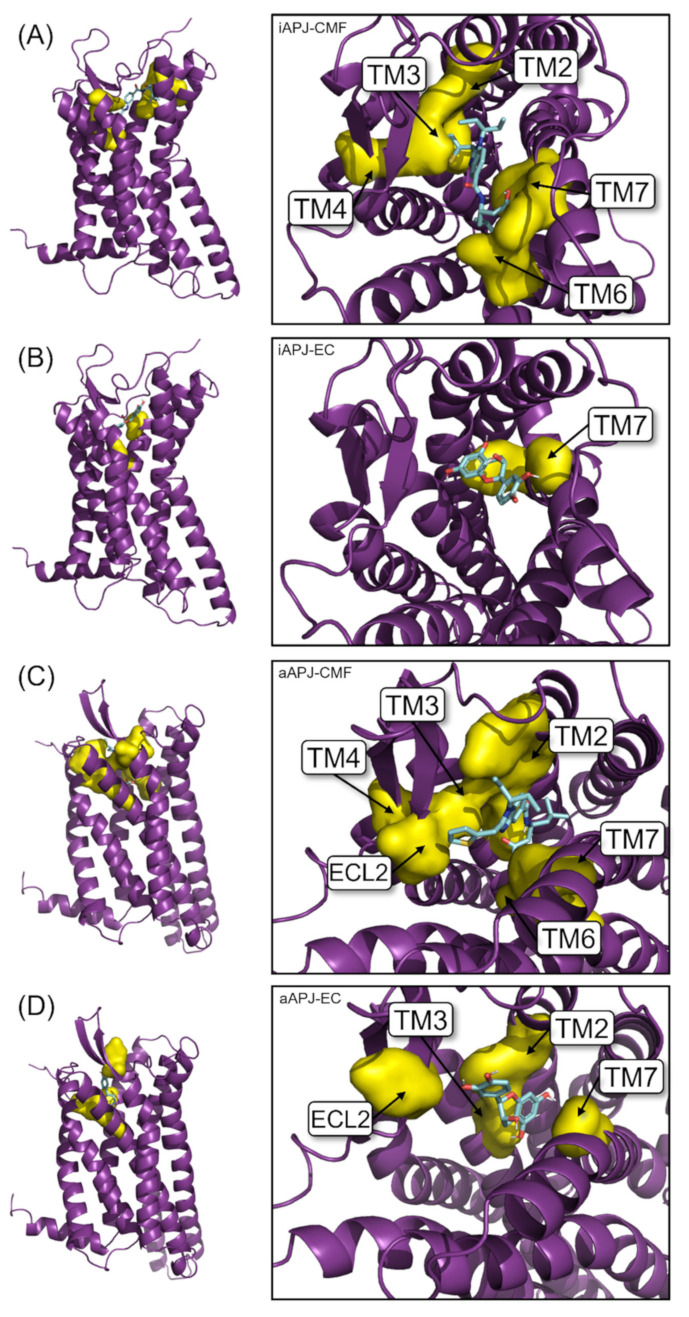
On the left side of each model, the APJ receptor seen from the side is represented in 3D, and on the right, the same receptor, but seen from above. Yellow shows the surface of the residues with which the ligand is in contact, and the arrows indicate the transmembrane domain (TM) to which the residue corresponds. (**A**) The interaction of CMF-019 with the receptor in its inactive conformation: it is observed how the ligand has contact with TM2, 3, 4, 6, and 7. (**B**) The interaction of (-)-epicatechin with the receptor in its inactive conformation: it is observed how it only has contact with the TM7. (**C**) The interaction of CMF-019 with the receptor in its active conformation: it is observed how the ligand has contact with TM2, 3, 4, 6, and 7, and with the extracellular loop (ECL) 2. (**D**) The interaction of (-)-epicatechin with the receptor in its active conformation: it is observed how it has contact with the TM2, 3 and 7, and with the ECL2.

Figure 4 shows the interactions between EC and CMF-019 for both states (active and inactive) and the apelin receptor, a calculated mean binding affinity of −8.2 and −9.0, respectively, for the active state −7.3 and −8.1, respectively, for the inactive conformation (Table 4). In Figure 5, the binding site of each ligand in a three-dimensional representation is shown. CMF-019 in both conformations (active and inactive) interacts with five transmembrane (TM) domains. EC in the active conformation interacts with three TM domains and in the inactive conformation only with one domain; these differences could cause the receptors’ structural modifications that are seen later with the analysis of the hydrogen bonding formed between the residues Tyr221 and Tyr309. Both ligands (Figure 6) in the active conformation of the receptor can interact with a residue of the extracellular loop 2 (ECL2).

**Figure 7:**
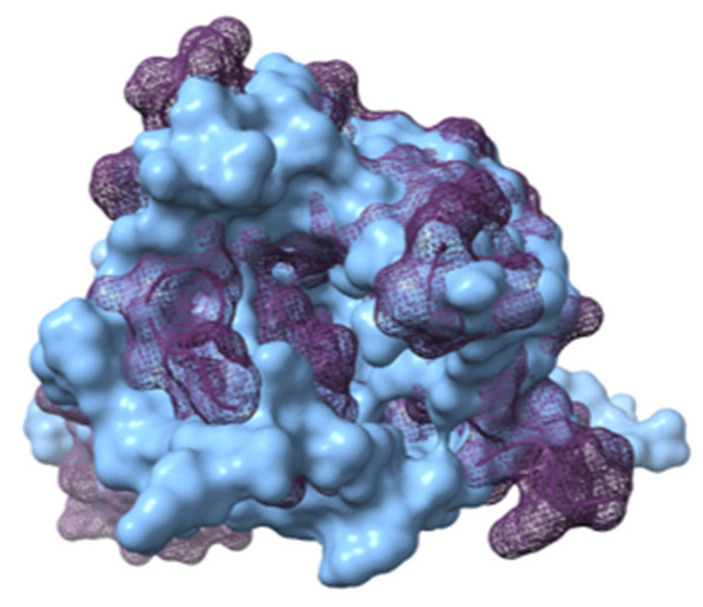
APLNR in both conformations superimposed. Active on purple mesh and inactive on blue surface.

For both APLN states, the cavity where the binding site is found has different sizes. With the solvent-accessible surface area/volume analysis (SASA), performed in both conformations with the GROMAS algorithm *gmx sasa*, for the iAPLNR the cavity area of 1190.86 Å^2^ and a volume of 1639.88 Å^3^ were calculated. For the aAPLNR, the area measured was 718.398 Å^2^ with a volume of 582.251 Å^3^. In Figure 7, we can see in blue (iAPLNR) and purple (aAPLNR) the superposition of both conformational states of the receptor and visualize in another way the difference in the available space in each receptor conformation, and also visualize how, for a ligand, it would be easier to interact with a greater number of amino acids if the distances between the transmembrane domains were smaller, as in the iAPLNR-EC case, that only has contact with the TM7. On the other hand, for the aAPLNR, the EC enters into contact with three transmembrane domains (TM2, 3, and 7) in addition to ECL2 (Figure 6).

### 2.6. Molecular Dynamics (MD)

Six MD simulations were performed: (1–2) a simulation for each receptor’s status alone (aAPLNR and iAPLNR), and (3–6) a simulation of each ligand (EC and CMF-019) in complex with each receptor conformation (aAPLNR and iAPLNR). The root mean square deviation (RMSD) from all trajectories was calculated using the protein backbone from the first frame as a template. The hydrogen bonds formed between the ligands and the protein were calculated.

#### 2.6.1. RMSD Analysis

**Figure 8:**
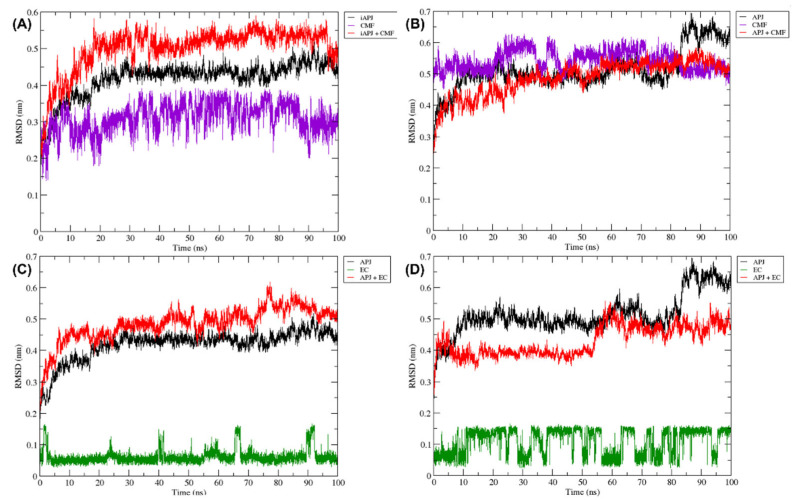
The RMSD plots were obtained from a 100 ns MD simulation. On the ordinate axis, the variation in distance is shown in nanometers and on the abscissa axis, the trajectory time is shown in nanoseconds. In all quadrants, we can see the movement of the APLN receptor alone (black line), the APLN receptor in complex with a ligand (red line), the CMF ligand only in (**A**,**B**) (purple line), and the EC ligand only in (**C**,**D**) (green line). iAPLNR = inactive APLNR; aAPLNR = active APLNR; EC = (-)-epicatechin; CMF = CMF-019; APJ = APLNR; black line = receptors MD simulation; red line = receptor in complex with a ligand; green = EC within the receptor; purple line = CMF-019 within the receptor; ns = nanoseconds; nm = nanometers.

The RMSD variation was calculated with the GROMACS algorithm “gmx rms”. The structure variation in iAPLNR and aAPLNR remains stable throughout the trajectory, except for the last nanoseconds in the active conformation, which increases by ~0.2 nm. The iAPLNR + CMF-019 complex is stable and does not have sudden changes in the entire trajectory (Figure 8A). The aAPLNR + CMF-019 complex is more stable since the analysis showed only a slight fluctuation (~0.15 nm) in the distances range (Figure 8B). On the other hand, the EC in complex with iAPLNR does not present significant fluctuations during the entire simulation (Figure 8C); however, in the aAPLNR + EC complex analysis, a sudden change at ~60 ns, causing an alteration of ~0.1 nm in the RMSD (Figure 8D), is induced.

#### 2.6.2. Hydrogen Bonds Analysis

**Figure 9:**
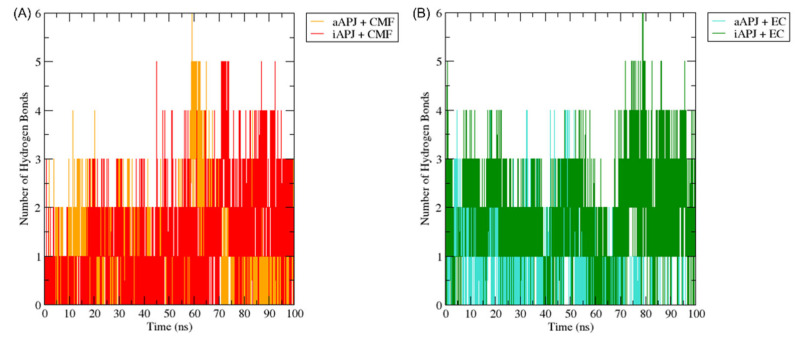
The H-bond plot shows the evolution of all hydrogen bonds formed by (-)-epicatechin (EC) and CMF-019 (CMF) in complex with the iAPLNR and aAPLNR, during a 100 ns MD simulation. For both graphs, on the ordinate axis, we see the number of hydrogen bonds, and on the abscissa axis, we have the simulation time expressed in nanoseconds. (**A**) The CMF can form up to six hydrogen bonds in the active conformation, and five in the inactive one of the APLN receptor during the entire trajectory, with an average of approximately three hydrogen bonds for both conformations. (**B**) EC manages to form up to five hydrogen bonds in the active conformations and up to six hydrogen bonds in the inactive conformation. For the active conformation of the receptor (aAPLNR), the hydrogen bonds begin to disappear in the last ~30 ns of the simulation, until only one is formed. This is because in the presence of EC, the APLNR modifies its conformation after a period of time—the same phenomenon that we observed with the RMSD of the EC + aAPLNR complex. ns = nanoseconds.

The hydrogen bond formation was calculated with the GROMACS algorithm “gmx hbond” over the six simulations. The hydrogen bonds formed by CMF-019 in iAPLNR and aAPLNR (Figure 9A) and the hydrogen bonds formed by EC in both receptor states (Figure 9B) suggest a high-affinity binding process for both ligands throughout the trajectories. This phenomenon occurs because water fills all protein cavities, which confers more stability to the complex [40].

### 2.7. Molecular Features Pointing to Biased Agonism (β-Arrestin Pathway)

#### 2.7.1. Hydrogen Bond between Tyr221 and Tyr309

**Figure 10:**
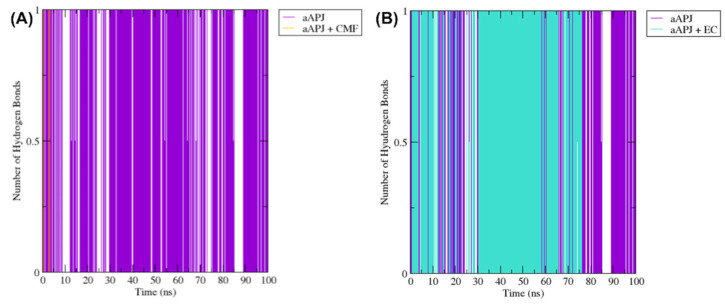
The H-bond plot shows the evolution of the hydrogen bonds formed by the TM5 Y221 residue and the TM7 Y309 residue. (**A**) When the receptor is alone (aAPLNR—purple lines), we see how these two residues form the hydrogen bond in practically the entire trajectory. However, when CMF-109 (CMF) binds, it induces a conformational change in the receptor that causes a gap between the TM5 and TM7, thus preventing the formation of the hydrogen bond. (**B**) On the other hand, when (-)-epicatechin (EC) is inside, it allows the receptor to maintain the spatial arrangement so that these two tyrosine residues are close enough to form the hydrogen bond at least until nanosecond ~75, which is when CE begins to induce a conformational change in the receptor, as we had observed in the RMSD and protein–ligand hydrogen bond formation analysis. APJ = APLNR; ns = nanoseconds.

The hydrogen bond formation between Tyr221 in transmembrane domain 5 (TM5), and the Tyr309 in the TM7, have been identified as crucial for β-arrestin recruitment. Suppose this hydrogen bond is not formed, and the transmembrane domains cannot interact with each other. In that case, the opening of an intracellular cavity where the G protein can dock is possible [41]. So, using the GOMACS “gmx hbond” algorithm, we calculate the hydrogen bonds formed in the simulated trajectories between these two tyrosine residues. The hydrogen bond is only formed in the aAPLNR (violet lines, Figure 10). In the presence of CMF-019, these residues form hydrogen bonds in a limited manner (mustard yellow lines in Figure 10A). In the presence of EC, the residues (Y221 and Y309) form hydrogen bonds in almost all of the MD simulations until the nanosecond ~70 (turquoise blue lines in Figure 10B), suggesting an EC-induced receptor rearrangement to a conformation where β-arrestin is not able to be recruited.

On the other hand, it has been suggested that for the optimal formation of hydrogen bonds, the distance of a donor or an acceptor must be between 2.7 Å and 3.3 Å from another acceptor or donor [40,42]. We calculated the distance between the Y221 and Y309 residues using the VMD software [43]. In the presence of EC (Table 5), the average distance values were 2.97 Å when the donor is the Tyr221 and the acceptor is the Tyr309, and 2.94 Å when the donor is Tyr309 and the acceptor is Tyr221. In the presence of CMF-019, the average distance value was 6.11 Å when the donor is Tyr221 and the acceptor is TyR309, and 6.02 Å when the donor is Tyr309 and the acceptor is Tyr221. These results suggest that the distances between both residues in the presence of CMF are not optimal for establishing hydrogen bonds, and in the case of the EC, they are optimal for the formation of the hydrogen bond, strongly suggesting that EC can recruit β-arrestin.

#### 2.7.2. Cluster Analysis and Clash with G Protein

To search for more evidence showing that EC induced a receptor structural rearrangement to promote this β-arrestin recruitment, we analyzed the preferred β-arrestin or G protein recruitment in the active states of ligand–protein complexes using the “gmx cluster” algorithm to calculate the clusters formed throughout the aAPLNR DM simulation in complex with CMF-09 and EC. The analysis was established with a 0.2 nm cutoff with 10,000 frames of the complete trajectory (100 ns), and all of the ligand–protein complex conformations that remained within the 0.2 nm range were considered as being in the same cluster.

**Figure 11:**
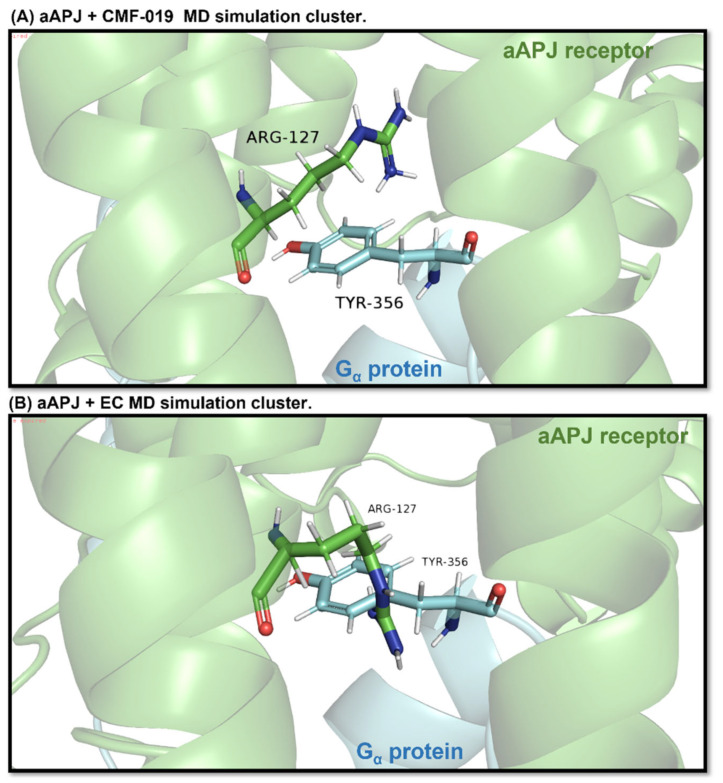
In the images, the aAPLNR and the same receptor residue Arg127 are shown in green. Cyan blue shows the G protein and the same protein residue, Tyr356. (**A**) It can be seen how, in the simulation, the aAPLNR in complex with CMF-019 is at a sufficient distance to allow the G protein to enter the cavity. (**B**) On the other hand, the aAPLNR in complex with the EC is in such a conformation that when placing the G protein, the Tyr356 residue collides spatially with the Arg127 residue of the receptor, which would indicate that the G protein cannot be attached in the closed cavity.

The obtained clusters were superimposed on the muscarinic acetylcholine receptor 1-G11 protein complex (PDB ID: 6OIJ) (used as a template) with the Chimera software [44]; then, the muscarinic-1 receptor was removed from the model, leaving the G protein coupled to APLNR for visual analysis within the receptor cavity (Figure 11).

### 2.8. Binding Free Energy Calculations (MM/GBSA)

Using the MM/GBSA method, we calculated the average binding free energies (∆G binding) of EC and CMF-019 in complex with iAPLN or aAPLN receptors. For the complete analysis, we used the 100-nanoseconds simulation (10,000 frames were considered). ∆G values of −32.25 ± 0.83 kcal/mol and −31.50 ± 1.11 kcal/mol for the CMF-019 in complex with aAPLNR and iAPLNR, respectively, were obtained. For EC, in complex with aAPJ and iAPJ, values of −11.61 ± 0.71 kcal/mol and −22.59 ± 0.76 kcal/mol, respectively, were obtained (Table 6). The energetic contribution of each residue to the overall free binding energy of ligand binding (EC and CMF-019) for both receptor conformations is shown in Table 7.

### 2.9. Effect of β-Arrestin Pathway Inhibition of Akt Phosphorylation

**Figure 12:**
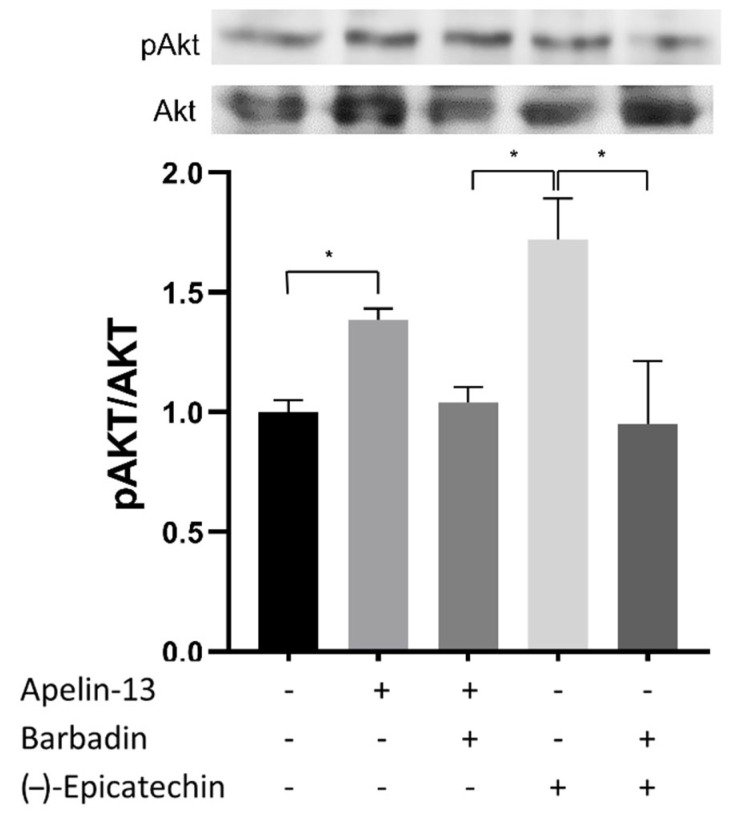
Acute effects (30 min) of apelin−13 and (-)-epicatechin (EC) on upstream Akt activation. These effects were blocked by the β-arrestin inhibitor, Barbadin, as shown in the representative blots (each Western blot is representative of three independent experiments). Data are expressed as mean ± SD (*n* = 3). * = *p* < 0.05.

Figure 12 shows the effect of the β-arrestin inhibitor, Barbadin, in the EC-induced Akt phosphorylation. All groups are normalized to the effect observed in the control group. A significant difference (*p* < 0.05) is shown in the Akt/pAKT effect between the apelin-13 and EC groups vs. the control group, as well as a difference in the effect between the EC group vs. Barbadin + EC.

## 3. Discussion

The main results reported in this work are:

(-)-Epicatechin binds specifically to an apelin receptor and epicatechin may act in a biased manner when activating APLNR.

The results showed a low *K_d_* value and a high *K_A_* value (1.755 × 10^−12^ M and 20.8, respectively), suggesting a very high affinity for the receptor [45]. Tau (the inverse of the fraction of receptors that the agonist must occupy to obtain half the maximum response) [37] is 21.8 for EC, pointing out the high efficiency of EC to bind to and produce a response at this receptor. So, in this case, the hillslope (*h*) was 0.2202, 0.3432, and 0.2134 for apelin-13, EC, and EC + ML221, respectively, and the receptor’s affinity for other ligands decreased. Although this model has been criticized for being unrealistic, its simplicity makes it a useful empirical model, since its use requires little a priori knowledge about the properties of the protein or ligand under study [46].

For the molecular docking analysis, it has been reported that CMF-019 interacts, similarly to apelin-13, mainly with Ser6-His7-Lys8 residues in a hydrophobic APLNR cavity, also highlighting the interactions with Asp168 and Tyr88 residues [47]. In the active form, the receptor is expected to have an “open” cavity, leaving more space and leading CMF-019 to reach the receptor site 1 (just as apelin-13, Ser6-His7-Lys8) [48]. Moreover, Phe291 seems to be an anchor for the ligand stabilization within the binding cavity; consequently, Trp85 and Ile109 favor the CMF-019 aliphatic chain interaction in the binding site. In the inactive receptor status, the interaction with Asp168 also plays an essential role in the G protein pathway activation; in this condition, the ligand could be performing a G protein preassembly, preparing the system to signal when the conformation turns active [49,50]. 

Interestingly, the nature of apelin-13 hydrophobic and aromatic termini fits the molecular characteristics of EC, making it a perfect candidate for mimicking some of the effects developed by apelin-13. In the first approach of EC binding to APLNR analysis, we found that it reaches a deeper site than the CMF-019 fitting site, which can also be occupied by apelin-13. The main interactions are Tyr182, Tyr264, Pro292, and Tyr299 [50]. Some of these amino acid residues in both APLNR states are related to the β-arrestin pathway [51]. In the same sense, the affinity of apelin-13 and EC for the APLNR active state is well represented by the ∆G values. These data suggest that CMF-019 is a better agonist than EC—at least ten times better. Nevertheless, CMF-019 and EC reach different places of the binding site 1 cavity, and a comparison between them is not necessarily suitable.

Regarding the ligand–receptor interaction, the hydrogen bonds formed in the binding process are characteristic of high-affinity binding. When a protein is in solution, the solvent—in this case, water—fills all of its cavities. When a ligand comes close to a binding site, it must wield a force strong enough to repel the force exerted by the water, conferring more stability to the complex [52]. So, the hydrogen bond formation analysis allows a closer look at the binding phenomenon. 

In the case of the aAPLNR + CMF-019 complex, no hydrogen bond between TM5 and TM7 transmembranal loops was expected, since CMF-019 is known to activate the G protein pathway selectively [47,53]. In addition, in the aAPLNR MD simulation, the hydrogen bonds between the two tyrosine residues of the TM5 and TM7 that are needed for the β-arrestin recruitment are formed [41], corroborating the idea that EC can recruit β-arrestin in the APLNR active state. In the aAPLNR+ EC MD simulation, the distance between the residues from the TM5 and TM7 (Tyr221 and Tyr309) essentially remains within the hydrogen bond formation range. The opposite happens in the case of aAPLNR + CMF-019 MD simulation, where the residues remain above 3.4 Å and, therefore, cannot form hydrogen bonds.

On the other hand, we determined whether Arg127, a residue that is part of the TM3 receptor intracellular cavity, is in a specific position that allows the G protein to enter into the receptor cavity without overlapping with the G protein Tyr356 residue. If these two residues clash, the G protein cannot dock, and the β-arrestin is recruited instead [41,54,55]. Interestingly, the “conformer” generated by CMF-019 allows an adequate G protein accommodation, since the Arg127 residue in the receptor does not overlap with the G protein Tyr356 residue. On the other hand, the induced receptor “rotamer” in the presence of EC shows how the residues collapse, meaning that the Gα-protein is not capable of loading. Therefore, these results and the analysis of the hydrogen bonds formed between intra-receptor tyrosine residues (Y221 and Y309) show that CMF-019 recruits G proteins and EC recruits β-arrestin, and strongly suggest a bias for EC binding to APLNR.

In general, all of the data reveal that all binding processes analyzed are favorable, supporting EC as a ligand to the APLNR. EC induces a conformational change in the receptor after it has promoted the β-arrestin recruitment. On the other hand, the EC-induced Akt phosphorylation is mediated mainly by activating GPCRs capable of recruiting β-arrestin, since this effect decreases when a specific inhibitor, Barbadin, blocks the β-arrestin pathway (Figure 12).

## 4. Materials and Methods

### 4.1. Protein Isolation from Mouse Skeletal Muscle

To isolate (-)-epicatechin-interacting proteins from skeletal muscle, we synthesized an affinity column according to a previously reported method [7]. For the isolation, a homogenate of C57BL/6 mice quadriceps was incubated overnight (4 °C) with the affinity chromatography column; noninteracting proteins were eliminated after several column washes (10×) with PBS (1×, pH = 7.4), and interacting proteins were detached with an acidic solution (MES, pH = 4.5). 

SDS-PAGE separated isolated proteins. We analyzed, by mass spectrometry, those bands corresponding to a GPCR typical molecular weight (40–70 KDa); the eluted gel was cut and digested with trypsin, and RP-HPLC separated generated peptides, and mass spectrometry (ESI/MS-MS) was run for identification. Then, an in-depth analysis was carried out, comparing the peptides with the database of the ProteinLynx Global SERVER (PLGS) program. 

### 4.2. Akt Phosphorylation in C2C12 Assay

A total of 800,000 C2C12 cells (ATCC^®^, Manassas, VA, USA, CRL-1772™) were cultured on 60 cm2 Petri dishes in DMEM-F12 culture medium supplemented with 10% FBS and 1% antibiotics. The cells were incubated at 37 °C and 5% CO2 until 80% confluence was reached. Five groups of cultured cells (three dishes per group) were used; the groups were stimulated with (1) vehicle (control); (2) 1 μM of EC; (3) 1 μM of APLNR antagonist (ML221) (Cayman, Ann Arbor, MI, USA) for 30 min, then 30 min with 1 μM EC; (4) 1 μM of GPER antagonist (G15) for 30min, then 30 min with 1 μM EC; and (5) 1 μM of ML221 and 1 μM of G15 for 30 min and then 30 min with 1 μM EC. 

The cells were treated with 0.05% trypsin to extract the proteins with RIPA buffer. The total protein content was quantified using the Bradford method. SDS-PAGE was performed in which 60 ug of protein was loaded to subsequently analyze the phosphorylation (activation) of Akt by Western blot using Akt1,2,3 and pAkt1,2,3 antibodies (Abcam, Cambridge, UK, ab126811, and ab183758).

Another set of experiments was performed using Barbadin 19.1 μM (AOBIOUS, Gloucester, MA, USA, cat. #AOB37364) and a specific β-arrestin activation blocker.

### 4.3. β-Arrestin Recruitment Assay

To determine the β-arrestin recruitment in vitro, the PathHunter^®^ eXpress GPCR kit was used (Eurofins 93-0250E2CPM0M, Santa Clara, CA, USA). The receptor is tagged by incorporating a short β-galactosidase ProLink amino acid sequence into its C-terminus and is stably expressed in the Chinese hamster ovary cell line (CHO-K1). Likewise, β-arrestin-2 is marked with the other enzyme, β-galactosidase acceptor fragment, expressed in this cell. Thus, the union of the ligand with the labeled receptor will attract the β-arrestin and complement the activity of the β-galactosidase enzyme, which, when stimulated with the substrate, will be able to metabolize it to give a chemiluminescence response. Serial dilutions of agonists and antagonists were prepared with the cell-coating reagent; after incubation for 1 h, the working detection solution was poured (substrate for the galactosidase enzyme) and incubated in the dark for 1 h. The reaction was then read in a standard luminescence plate reader (Biotek^®^ PowerWave XS, Winooski, VT, USA).

### 4.4. G Protein Pathways in EPI-Induced Activation of APLNR

To determine the participation of G protein-initiated pathways in the EPI-induced APLNR stimuli, we employed the cAMP Hunter eXpress GPCR assay (Eurofins 95-0147E2CP2M). The signaling involves a membrane-bound adenylate cyclase. Gαi-coupled to APLNR modulate cAMP by inhibiting adenylate cyclase. In this case, the Chinese hamster ovary cell line (CHO-K1) expressing APLNR coupled to Gαi is stimulated using forskolin and dose–response curves to agonist-activating Gαi.

### 4.5. Molecular Docking

EC and molecules of interest were downloaded as “mol2” from the ChemSpider online molecular database (http://www.chemspider.com, accessed on 20 October 2020), and converted to “PDB” in PyMol and then processed using AutoDock Tools 4.0 [56], where polar hydrogens and Gasteiger charges were added. Potential receptors in their active and inactive conformations were downloaded from the online GPCR database (https://gpcrdb.org, accessed on 20 October 2020), and then processed in AutoDock Tools version 4.0 to add polar hydrogens and *Kollman charge*. The grid box for a blind docking assay was placed in the receptor center with coordinates at x = 24.096, y = 62.263, and z = 11.917; dimensions of x = 50, y = 60, and z = 50 were obtained based on the center of the protein. Following this, 1000 independent replicates were performed with the Vina software [57] using a previously described script written in Shell [58] to facilitate the work with each ligand. Then, based on the ligand coordinates, the receptor-binding site was calculated, and the most frequent interaction site was defined and established as the most favorable conformation.

### 4.6. Molecular Dynamics

The ligand topology was generated on the CGenFF online server (https://cgenff.umaryland.edu/, accessed on 1 November, 2020). For the MD simulations and the topology generation of the receptors, GROMACS 5 and the CHARMM36-Jul2020 force field were used [59]. The ligand–protein complex was assembled within a dodecahedron with a minimum distance of 1 nm from the protein edge and with periodic boundary conditions. The ligand coordinates were the same as the docking assay previously performed. The TIP3P model was used for solvation with water, and then some water molecules were replaced by 0.15 M NaCl, including those necessary counterions for protein neutralization. Having already solvated the systems, minimization was carried out using the steep descent function for 50,000 steps with a maximum force of 10 kJ/mol. The complexes were then equilibrated with the NVT ensemble for 100 ps, followed by equilibration with the NPT ensemble for another 100 ps; in both cases, the protein and the ligand position were restrained. Finally, the dynamic productions were carried out in the NPT ensemble at 300 K, 1 atm pressure, and 100 ns; the V-rescale temperature coupling method and the Parrinello–Rahman coupling method were used. The Ewald particle mesh method was used to calculate the long-range electrostatic interactions. The leap-frog algorithm calculated the motion equation with a two-fs time step. 

### 4.7. Binding Free Energy Calculations

With the use of molecular mechanics with generalized Born surface area (MM/GBSA) algorithms [60,61], we predicted the interaction energy between the ligand and receptor to estimate the free binding energy between the receptor and the EC. For this, we used the Gmx_MMGBSA algorithm, and MM-GBSA analysis was performed based on three structure systems: the protein, the ligand, and the complex ligand protein. 

## 5. Conclusions

(-)-Epicatechin is capable of binding to the receptor with specificity to preferentially activate it towards the β-arrestin pathway. In other words, the particular specificity of (-)-epicatechin binding to the receptor induces a conformational change that promotes β-arrestin recruitment (demonstrated with in silico and in vitro models), and with this we can propose that (-)-epicatechin works as a bias agonist.

## 6. Perspectives

In the present work, evidence from several in silico and in vitro experiments is presented that suggests (-)-epicatechin is a biased agonist of APJ; in order to corroborate this finding and provide more evidence of this phenomenon and the importance of it on skeletal muscle functionality, more studies are needed. We suggest prospective in-vitro assays to show how APLNR-biased agonism is involved in processes relevant to skeletal muscle function/regulation, such as performance and differentiation.

## Figures and Tables

**Table 1:** Reported effects of EC-interacting isolated receptors on skeletal muscle.

Protein	Function in Skeletal Muscle
Apelin receptor	Apelin signaling through APJ enhances muscle function by triggering mitochondriogenesis, autophagy, and anti-inflammatory pathways in myofibers as well as enhancing the regenerative capacity by targeting muscle stem cells. It mirrors the effects of maternal exercise on mitochondrial biogenesis and fetal muscle development. It also promotes non-shivering thermogenesis gene expression in fetal and offspring muscle impaired due to maternal obesity, which intergenerationally protects offspring from diet-induced obesity and metabolic disorders [17,18,19,20]
Cation-dependent mannose-6-phosphate receptor	Not reported
Gamma-aminobutyric acid receptor subunit beta-2	GABAergic receptors contribute to the cardiovascular responses during the activation of group III/IV skeletal muscle afferent fibers [21]. Modulates sympathetic vasomotor outflow and the pressor responses to activation of metabolically sensitive skeletal muscle afferent fibers, and it also assists the development of motor circuit activity, motoneuron survival, and muscle innervation [12,22].
Glutamate receptor ionotropic, NMDA 2A, and glutamate receptor ionotropic, NMDA 2D	NMDA receptor is found in muscle differentiation, promotes Ca^2+^ influx in myoblasts, thereby triggering myoblast fusion. It mediates the “nerve–muscle” system, due to the enhancement of NO synthesis under the influence of a glutamate. It promotes acceleration of motor neuron development and is directly involved in skeletal muscle maturation. Exercise triggers NMDA signaling in motor neurons. May accelerate the development of the motor units [13,14,15,23].

**Table 2:** Comparative effects of APJR stimuli and reported (-)-epicatechin-induced effects on skeletal muscle. ↑= increased values.

Apelin		(-)-Epicatechin	
Experiment Models	Pathway or Molecule	Experiment Models	Pathway or Molecule
Developing cardiovascular in mouse, xenopus, and zebrafish embryos	↑MEF2 [24]	Hindlimb muscles from exercised C57BL/6 mice.	↑MEF2 [25]
Chow-fed rat triceps	↑PGC-1β, ↑activity of cytochrome C oxidase [24]	Old and trained C57BL/6N mice	↑PGC-1β, ↑activity of cytochrome C oxidase [26]
In HFD mice	↑PGC-1α, TFAM [27]	C57BL/6 mice quadriceps	↑PGC-1α, TFAM [28]
Myocardial microvascular endothelial cells (MMVEC)	↑eNOS [29]	Human coronary artery endothelial cells (HCAEC)	↑eNOS [30]
In HFD mice	↑PGC1α, AMPK [27]	Human quadriceps	↑PGC1α, AMPK [31].
C2C12 skeletal myotubes	↑Akt phosphorylation [32]	C2C12 skeletal myotubes	↑Akt phosphorylation [33]
Obese and insulin-resistant mice	↑Mitochondrial biogenesis, PGC1α, NRF-1, TFAM [24]	Human muscle biopsies from quadriceps femoris	↑Mitochondrial biogenesis, PGC1α, NRF-1, TFAM [34]
Muscle cells from young and aged human donors	↑mTOR, and P70S6K, Akt phosphorylation [17]	Male CD-1 mice.	↑mTOR, and P70S6K, Akt phosphorylation [35]
Male Wistar rats	↑Activity of Citrate Synthase [19]	C2C12 skeletal myotubes	↑Activity of Citrate Synthase [5]
Different rodent models	↑UCP3 [24]	Male ICR mice	↑UCP3 [36]

**Table 3:** Reported effects of EC-interacting isolated receptors on skeletal muscle.

Ligand	IC50	Tau (τ)	*K_A_*	*Bmax*	*Kd*	h
Apelin	-	-	-	801,286	2.508 × 10^−11^ M	0.2202
EC	-	21.8	20.8	810,100	1.755 × 10^−12^ M	0.3432
EC + ML221	6.93 × 10^−11^ M	1.53	0.53	733,981	1.012 × 10^−9^ M	0.2134

**Table 4:** Summary of the data obtained from the molecular docking assay of EC and CMF-019 against iAPLNR and aAPLNR. ∆G values are presented as mean ± standard deviation.

Protein	Ligand	∆G	Residue Interaction
Active APLNR	EC	−8.2 ± 0.005	Trp85, Ile109, Tyr182, Pro292
	CMF-019	−9 ± 0.276	Trp85, Tyr88, Ile109, Arg168, Met183, Tyr264, Phe291
Inactive APLNR	EC	−7.3 ± 0.028	Pro292, Tyr299
	CMF-019	−8.1 ± 0.315	Trp85, Ile109, Arg168, Tyr271, Met288, Phe291

**Table 5:** Distances in Å (Armstrong) between the TM5 Y221 residue and the TM7 Y309 residue throughout all the aAPLNR complex MD simulations. ns = nanoseconds; O = oxygen acceptor; H = hydrogen donor.

	EC		CMF	
Trajectory	Tyr309O-HTyr221	Tyr221O-HTyr309	Tyr309O-HTyr221	Tyr221O-HTyr309
10 ns	3.04	2.22	4.15	3.96
20 ns	3.41	2.31	3.85	3.57
30 ns	3.08	2.11	3.42	3.38
40 ns	4.25	3.41	4.66	3.99
50 ns	2.7	2.94	4.85	4.06
60 ns	2.18	2.8	5.52	4.68
70 ns	2.15	3.07	10.31	11.13
80 ns	3.49	4.34	6.42	5.72
90 ns	3.3	3.29	9.31	9.95
100 ns	2.12	2.92	8.67	9.82

**Table 6:** Summarized data from the MM/GBSA method. vdW: van der Waals; elect: electrostatic; solv: solvent.

Protein	Ligand	Calculated Free Energy of Decomposition (kcal/mol)				
		∆G Binding	∆E vdW	∆E Elect	∆G Gas	∆G Solv
Active APLNR	EC	−11.61 ± 0.71	−19.41 ± 0.75	−9.35 ± 1.59	−28.76 ± 1.92	17.15 ± 1.50
	CMF	−32.25 ± 0.83	−46.50 ± 0.85	−27.89 ± 1.84	−74.38 ± 2.24	42.14 ± 1.81
Inactive APLNR	EC	−22.59 ± 0.76	−31.47 ± 0.57	−39.02 ± 2.55	−70.49 ± 2.55	47.15 ± 2.06
	CMF	−31.50 ± 1.11	−43.08 ± 0.64	−13.69 ± 2.28	−56.77 ± 244	25.27 ± 1.56

**Table 7:** Ligand and residue energetical contribution to global free binding energy calculated by MM/GBSA decomposition.

Receptor	Ligand	Residue	∆G Binding	Std. Err.
Active APLNR	EC	ILE 8	−0.0746	0.0199
		TYR 12	−0.0143	0.0091
		PHE 55	−0.0029	0.0043
		LEU 59	−0.0143	0.0050
		TRP 62	−0.2029	0.0805
		TYR 65	−0.3447	0.1151
		THE 66	−0.2534	0.0692
		ASP 69	−0.0297	0.0514
		TYR 70	−0.3177	0.0821
		SER 83	−0.0045	0.0053
		ILE 86	−0.0318	0.0213
		PHE 87	−0.0024	0.0034
		ARG 145	−0.0685	0.0400
		CYS 158	−0.3188	0.0918
		TYR 159	−0.2368	0.0779
		MET 262	−0.6250	0.1350
		PHE 265	−0.2304	0.0678
		PRO 266	−0.1681	0.0603
		THR 269	−0.0327	0.0183
		TYR 273	0.0056	0.0020
		EC	−4.6858	0.3731
	CMF	ILE 8	−0.0144	0.0037
		TYR 12	0.0815	0.0190
		LEU 59	−0.0177	0.0148
		TRP 62	−0.6329	0.1180
		TYR 65	−0.2503	0.0843
		THR 66	−0.0272	0.0273
		ASP 69	0.0094	0.0062
		TYR 70	−0.2867	0.0614
		ILE 86	−0.4727	0.0809
		PHE 87	−0.2232	0.0742
		ARG 145	−0.7602	0.1734
		CYS 158	−0.3870	0.0974
		TYR 159	−0.6344	0.1119
		MET 160	−0.1602	0.0540
		TYR 240	−0.3717	0.0971
		LYS 243	−1.4040	0.1433
		TYR 246	−1.7127	0.1099
		LEU 261	−0.8716	0.0898
		MET 262	−1.5429	0.0996
		ASN 263	0.0067	0.0056
		PHE 265	−2.0900	0.1014
		PRO 266	0.0297	0.0364
		THR 269	−0.1933	0.0418
		CMF	−17.9118	0.4758
Inactive APLNR	EC	ILE 14	−0.0112	0.0054
		TYR 18	0.0087	0.0078
		TRP 68	−0.0609	0.0573
		TYR 71	−0.0291	0.0292
		THR 72	−0.0155	0.0151
		TYR 76	−0.1145	0.0398
		SER 89	−0.0038	0.0017
		ILE 92	−0.0270	0.0132
		PHE 93	−0.0593	0.0196
		ARG 151	0.0188	0.0218
		LEU 270	−0.2012	0.0304
		MET 271	−2.6193	0.1652
		ASN 272	−0.0825	0.0170
		PHE 274	−1.3984	0.0695
		PRO 275	−0.3658	0.0470
		THR 278	−0.0826	0.0428
		TYR 282	−0.0362	0.0480
		EC 316	−10.7886	0.4734
	CMF	ILE 14	−0.0770	0.0092
		TYR 18	0.1837	0.1171
		TRP 68	−2.1810	0.2573
		TYR 71	−1.4010	0.3388
		THR 72	−0.1017	0.0404
		TYR 76	−1.1871	0.1812
		SER 88	−0.1652	0.0353
		SER 89	−0.4662	0.1090
		TYR 90	−0.0075	0.0091
		ILE 92	−0.9967	0.1781
		PHE 93	−0.2054	0.0779
		VAL 147	−0.0611	0.0122
		ARG 151	−0.8365	0.4741
		CYS 164	−0.6322	0.1208
		TYR 165	−0.2165	0.0673
		MET 166	−0.0406	0.0267
		TYR 168	0.0008	0.0158
		GLU 181	0.4742	0.0621
		GLY 185	−0.0146	0.0146
		TYR 247	−0.0855	0.0354
		VAL 250	−0.0690	0.0356
		LYS 251	0.0795	0.1024
		TYR 254	−0.3275	0.1917
		MET 255	−0.0609	0.0648
		LEU 270	−0.2245	0.1747
		MET 271	−0.6861	0.1217
		ILE 273	−0.0747	0.0065
		PHE 274	−1.3556	0.2144
		PRO 275	−1.2212	0.0936
		THR 278	−0.9633	0.1438
		TYR 282	−0.0507	0.1013
		CMF	−16.2988	0.8901

## Data Availability

The data are available upon request.
